# 

**Published:** 1906-11-10

**Authors:** 


### NOTICE TO CORRESPONDENTS.

All MSS., Letters, Books for Review, and other matters intended for the Editor should be addressed The Editor, "The Hospital," The Hospital Buildings, 28 & 29 Southampton Street, Strand, W.C.

The Editor cannot undertake to return rejected MSS., even when accompanied by stamped directed envelope.

**Notice to Advertisers and Subscribers.**

All Advertisements, Orders for Copies of The Hospital, and Business Communications should be addressed to **THE MANAGER (Not to the Editor),** The Hospital, 28 & 29 Southampton Street, Strand London, W.C.

The Cost of Subscription, post free, is as follows:—Per week, 3½d.; per quarter, 3s. 3d.; per half-year, 6s. 6d.; per annum, 13s. Foreign and Colonial:—Per annum, 19s. 6d., payable, in all cases, in advance.

**NOTICE.—Vol. XL. of "The Hospital" (April to September 1906), in handsome cloth binding will shortly be on sale at the office of this Journal price 10s. 6d. Cases for binding the Numbers contained in this Volume 2s. Index to Vol. XL., 3½d., post free.**

**The Monthly Part for November is now ready. Price 1s., by post, 1s. 3d.**

**MEDICAL ELECTRICITY AND THE LIGHT TREATMENT.**
By Kate Neale, Sister in Charge Light Department, Guy's Hospital. Illustrated.
2/6 net, 2/9 post free.

**ELEMENTARY TREATISE ON THE LIGHT TREATMENT FOR NURSES.**
By James H. Sequeira, M.D. Lond. Illustrated.
2/6 net, 2/9 post free.

**ART OF FEEDING THE INVALID.**
By a Medical Practitioner and a Lady Professor of Cookery.
New and Popular Edition, with Special Chapter on Feeding in Consumption. 1/6 post free.

**DIET IN SICKNESS AND IN HEALTH.**
By Mrs. Ernest Hart. With an Introduction by Sir Henry Thompson, F.R.C.S., M.B. Lond.
3/6 post free.

**LECTURES UPON THE NURSING OF INFECTIOUS DISEASES.**
By F. J. Woollacott, M.A., M.D.
2/6 net, 2/9 post free.

**HINTS TO NURSES ON TROPICAL FEVERS.**
By S. F. Pollard, Sister Army Nursing Reserve, late Sister Guy's Hospital.
The Work is based upon the Personal Experience of the Author Abroad.
1/6 net, 1/8 post free.

**NURSING IN DISEASES OF THE THROAT, NOSE AND EAR.**
By P. Macleod Yearsley, F.R.C.S. Eng., M.R.C.S., L.R.C.P. Lond. Illustrated.
2/6 post free.

**OPHTHALMIC NURSING.**
By Sydney Stephenson, M.D., F.R.C.S.E.
New and Revised Edition, profusely Illustrated, and a Glossary of Terms.
3/6 net, 3/10 post free.

**THE MODERN NURSING OF CONSUMPTION.**
By Dr. Jane H. Walker.
1/- net, 1/2 post free.

**OUTLINES OF INSANITY.**
**A Popular Treatise on the Salient Features of Insanity.**
By Francis H. Walmeley, M.D. Popular Edition, stitched. 1/6 post free.

**SIMPLE INTRODUCTORY LESSONS ON MIDWIFERY.**
By M. Loane. Illustrated.
1/- net, 1/1 post free.

**INSTRUCTIONS TO MIDWIVES.**
Compiled by the Midwives Supervising Committee of the Corporation of Manchester.
6d. net, 7d. post free.

**SIMPLE SANITATION:**
**The Practical Application of the Laws of Health to Small Dwellings.**
By M. Loane. With Introductory Chapter on Sanitary Legislation by D. A. Mearns Proser. 1/- net, 1/2 post free.

**LECTURES ON HOME NURSING FOR THE POOR.**
By a District Nurse. Illustrated.
1/6 net, 1/8 post free.

**THE CARE OF CHILDREN.**
**Practical Hints for Mothers and Nurses at Home and Abroad.**
By Robt. J. Blackham, D.P.H. Lond., C.B.
Revised and Enlarged Edition.
1/6 net, 1/8 post free.

**THE HUMAN BODY:**
**Its Personal Hygiene and Practical Physiology.**
By B. P. Colton, Professor of Natural Science, Illinois University. Profusely Illustrated.
5/- post free.

***Catalogue of Nursing Manuals sent post free on application.***

**The Scientific Press, Ltd.,**

**Publishers of Nursing Text-Books, Charts, and Case-Books of all Descriptions.**

**28 & 29 SOUTHAMPTON STREET, STRAND, LONDON, W.C.**

